# Contemporary Ribonomics Methods for Viral microRNA Target Analysis

**DOI:** 10.3390/ncrna4040031

**Published:** 2018-11-09

**Authors:** Lauren A. Gay, Peter C. Turner, Rolf Renne

**Affiliations:** Department of Molecular Genetics and Microbiology, University of Florida, Gainesville, FL 32610, USA; lagay@ufl.edu (L.A.G.); pturner@mgm.ufl.edu (P.C.T.)

**Keywords:** KSHV, EBV, microRNA, CLIP, Ago, HITS-CLIP, PAR-CLIP, CLASH, qCLASH

## Abstract

Numerous cellular processes are regulated by microRNAs (miRNAs), both cellular and viral. Elucidating the targets of miRNAs has become an active area of research. An important method in this field is cross-linking and immunoprecipitation (CLIP), where cultured cells or tissues are UV-irradiated to cross-link protein and nucleic acid, the RNA binding protein of interest is immunoprecipitated, and the RNAs pulled down with the protein are isolated, reverse-transcribed, and analyzed by sequencing. CLIP using antibody against Argonaute (Ago), which binds to both miRNA and mRNA as they interact in RISC, has allowed researchers to uncover a large number of miRNA targets. Coupled with high-throughput sequencing, CLIP has been useful for revealing miRNA targetomes for the γ-herpesviruses Kaposi’s sarcoma-associated herpesvirus (KSHV) and Epstein-Barr virus (EBV). Variants on the CLIP protocol are described, with the benefits and drawbacks of each. In particular, the most recent methods involving RNA–RNA ligation to join the miRNA and its RNA target have aided in target identification. Lastly, data supporting biologically meaningful interactions between miRNAs and long non-coding RNAs (lncRNAs) are reviewed. In summary, ribonomics-based miRNA targetome analysis has expanded our understanding of miRNA targeting and has provided a rich resource for EBV and KSHV research with respect to pathogenesis and tumorigenesis.

## 1. Introduction

MicroRNAs (miRNAs) are short, non-coding RNAs of 21–23 nt that bind to target RNAs, inhibiting translation or inducing degradation or both [1]. The biogenesis of miRNAs involves transcription by RNA Pol II to generate a primary (pri-miRNA) 70–80 nt stem loop that is cleaved by Drosha within the nucleus. The resulting shorter precursor (pre-miRNA) is exported from the nucleus by Exportin5/Ran-GTP. Further processing by Dicer leads to a 21–23 nt RNA duplex. One strand becomes incorporated into RISC, the RNA-induced silencing complex, where it interacts with the target transcript to enable silencing [2]. The Argonaute (Ago) protein is an important component of RISC that facilitates the interaction between a mature miRNA and its target mRNA. Although there are four different variants of Ago in mammalian cells, only Ago2 is able to cleave the target transcript by its endonuclease activity [3].

The oncogenic gamma-herpesviruses Kaposi’s sarcoma-associated herpesvirus (KSHV) and Epstein-Barr virus (EBV) both express multiple miRNAs, 25 and 44, respectively [4]. The importance of miRNAs in cancer is underlined by the fact that several KSHV and EBV miRNAs have roles in processes associated with tumorigenesis [5,6]. In addition, miRNA expression levels are perturbed in several human cancers [7]. The KSHV miRNA miR-K12-11 is an ortholog of the cellular oncomiR miR-155 [8], and both miRNAs were found to induce human splenic B-cell expansion in a mouse model [9]. Moreover, in EBV-immortalized B cells, miR-155 is highly induced which points to the importance of this pathway [10].

Traditionally, miRNAs have been thought to bind via partial complementarity to sequences in the 3′UTR region of mRNA targets. Specificity of a miRNA for its target(s) was originally thought to be determined almost entirely by the seed sequence, the residues at positions 2–8 from the 5′ end of the miRNA [2]. Algorithms for predicting miRNA targets have relied heavily upon this model, although some take into account supplementary base-pairing at the 3′ end of the miRNA [11]. Newer experimental methods for target identification described below have challenged the importance of canonical seed-sequence binding, and revealed that many interactions between miRNAs and their targets occur via non-canonical binding events.

Investigation of interactions between miRNAs and RISC-associated RNAs with ribonomics methods that do not rely upon assumptions about seed sequences have also uncovered the existence of many long non-coding RNAs (lncRNAs) as potential miRNA targets [12]. The biological significance of miRNA–lncRNA interactions has been difficult to demonstrate in many cases as the lncRNAs are often of unknown function. However, in a few examples, miRNAs clearly can regulate lncRNA levels and appear to play roles in important biological processes such as regulation of cell cycle, metastasis, and stem cell differentiation [13].

## 2. CLIP and HITS-CLIP

The cross-linking and immunoprecipitation (CLIP) method and all of its descendants have in common the two components from which CLIP derives its name: crosslinking and immunoprecipitation. Initially, CLIP was developed in order to identify the RNA interacting partners of the neural protein Nova, which has been implicated in paraneoplastic neurologic degeneration [14,15]. CLIP has proved invaluable to the study of interactions between RNA-binding proteins (RBPs) and RNA, and methods derived from this have been used to study miRNA targets in both KSHV and EBV. What follows is a generalized description of the CLIP protocol based on the original experiment by Ule et al. [15]. Living cells are UV-irradiated at 254 nm to crosslink protein and bound nucleic acid. The cells are lysed and incubated with RNase to trim the RNA ends. After immunoprecipitation of the specific RBP of interest, the 5′ ends of the RNA are labeled with radioactive ATP and the 3′ adapter is added by ligation. The sample is run on a polyacrylamide gel to separate the desired RBP–RNA complexes from any other complexes present, and the material transferred to a nitrocellulose membrane. Free RNAs pass through, leaving protein and protein–RNA complexes on the membrane. The band of the expected size of the RBP–RNA complex of interest is excised, and bound material eluted and treated with proteinase K to release the bound RNA. Following dephosphorylation and 5′ adapter ligation [16], reverse transcription (RT) and PCR are performed and the resulting DNA fragments sequenced. In the first paper, 340 sequences were generated [15].

While sequencing capabilities were limited when the original method was established, high-throughput sequencing (HITS) was quickly incorporated into CLIP once the technology became available. Called HITS-CLIP, this improved protocol greatly expanded the amount of information which could be obtained from each experiment [17].

## 3. Ago-HITS-CLIP

**Figure 1:**
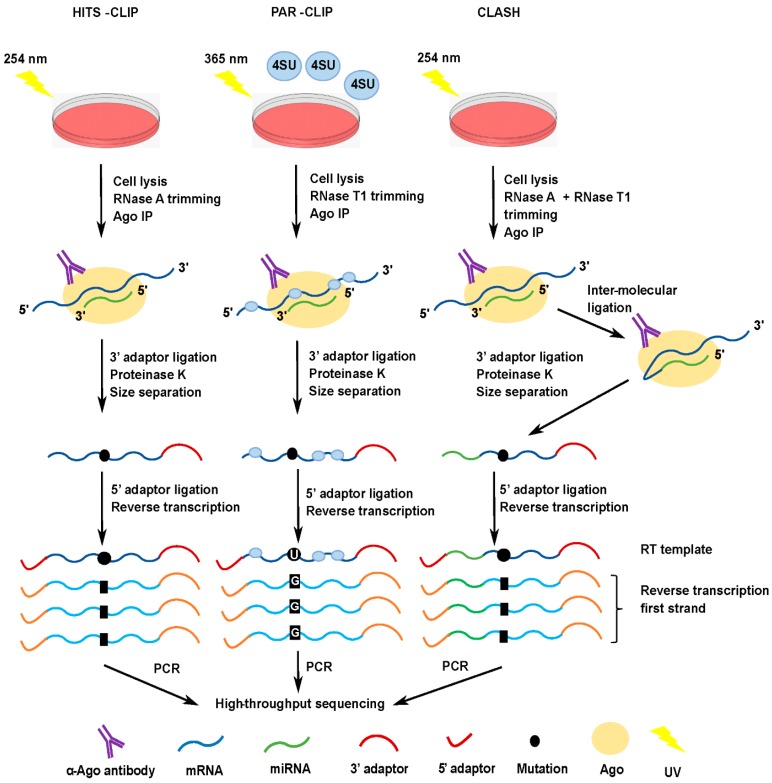
Outline of high-throughput sequencing cross-linking and immunoprecipitation (HITS-CLIP), photoactivatable-ribonucleoside-enhanced crosslinking and immunoprecipitation (PAR-CLIP) and cross-linking and sequencing of hybrids (CLASH) ribonomics protocols, with significant differences indicated. The steps from UV irradiation of cells through to sequencing library construction are shown. 4SU: 4-thiouridine; Ago: Argonaute. The black circles indicate mutations introduced by cross-linking damage, and the squares the nucleotide incorporated following reverse transcription. For PAR-CLIP, reverse transcription of RNA containing a crosslinked 4SU results in the misincorporation of a G in the opposite strand instead of A. The 3′ and 5′ adaptors are shown in red, and in orange following conversion to cDNA. Reproduced with permission from Sethuraman et al., *Nucleic Acids Research*; published by Oxford University Press, 2018, [12].

Previously, miRNA target studies were performed on a case-by-case basis for individual miRNAs [18]. A major advancement in the miRNA field came in 2009 when CLIP was first applied to study Argonaute in order to determine miRNA targetomes [19]. When Ago is pulled down, both species of RNA are brought with it (Figure 1). Sequencing libraries were made separately from the pooled miRNAs and pooled fragments of the target mRNAs. Sequencing and subsequent analysis enabled the identification of hundreds of potential interactions between miRNAs and their targets. This provided a genome-wide method for detecting miRNA targets in a given cell line or tissue.

Since the RNAs are separated from one another during the CLIP protocol, sequencing yields essentially two datasets: miRNAs and mRNAs. The two must then be assigned to each other bioinformatically. The mRNA reads are aligned with genomic sequences, and clusters are identified within transcripts. The field of putative miRNA targets is typically narrowed to those containing superclusters, which are clusters of reads that occur in multiple biological replicates. Three biological replicates are recommended. Based on the current models of how miRNA–mRNA interactions occur, most bioinformatics pipelines focused on 3′UTR regions of candidate genes that contain a perfect or near-perfect complement to the seed sequence (nucleotides 2–8) of the miRNAs [20]. One complication of this method was that a given supercluster can contain more than one miRNA seed sequence, which led to a significant false-discovery rate, determined by the fact that many targets could not be verified downstream by luciferase reporter assays or Western blotting [21].

To identify KSHV miRNA targetomes, a HITS-CLIP analysis was performed on BCBL-1 and BC-3 cells, two primary effusion lymphoma (PEL) cell lines latently infected with KSHV [21]. This early study revealed several new insights into KSHV miRNA biology. BC-3 cells were found to have much more abundant KSHV miRNAs compared to BCBL-1 cells, despite the fact that these two cell lines are both derived from B cells. In BC-3 cells, of the 30 most highly expressed miRNAs (cellular and viral), the top five are KSHV miRNAs, and 15 of the top 30 originate from KSHV. This raised the possibility that KSHV miRNAs may exert part of their effect on the host cell transcriptome by competing with human miRNAs in BC-3 cells. This displacement of host miRNAs from RISC may de-repress host genes under miRNA regulation. Analysis identified miRNA targets that were both known (e.g., BACH1) and previously unknown (vIL-6). Ago-HITS-CLIP resulted in lists of 1170 and 950 putative cellular KSHV miRNA targets from BCBL-1 and BC-3 cells, respectively. A small selection of targets was verified by making luciferase constructs with the 3′UTR downstream of the luciferase reporter. Individual miRNAs or miRNA clusters were expressed from plasmids that were co-transfected with the reporter plasmid, and in the majority of cases a significant decrease in luciferase activity was detected when the miRNAs were expressed. In a few cases, point mutations in the seed sequences in the 3′UTR were created, and in all cases, they resulted in loss of downregulation by miRNAs. Gene ontology revealed that miRNA targets were present in pathways of biological significance, including apoptosis, glycolysis, and lymphocyte activation.

Ago-HITS-CLIP has given us a greater understanding of targeting by miRNAs, and created a rich resource for the KSHV field. The limitations of Ago-HITS-CLIP, namely the assumptions that miRNAs will only be effective if they have a perfect or near-perfect seed sequence match within the 3′UTR of mRNA targets, may have resulted in failure to identify putative targets. However, although miRNAs and mRNAs may interact in this way much of the time, there has also been evidence that noncanonical base-pairing is significant as well [21]. Additionally, most analyses of Ago-HITS-CLIP data focused on potential targets solely within mRNAs, and did not take into account other classes of RNA. 

## 4. PAR-CLIP

A method related to HITS-CLIP was developed named PAR-CLIP: photoactivatable-ribonucleoside-enhanced crosslinking and immunoprecipitation [22]. Cells are grown in the presence of 4-thiouridine (4SU), a uracil ribonucleoside analog, which is incorporated into nascent RNA. Crosslinking is facilitated by UV irradiation at 365 nm. During this process, covalent bonds are formed between the 4SU and aromatic amino acids in close proximity within the Ago protein. From this point, the procedure is similar to HITS-CLIP, with Ago immunoprecipitation being followed by RNA isolation, library construction and sequencing (Figure 1). However, reverse transcription of the RNAs results in the misincorporation of a G opposite each crosslinked 4SU instead of A. After RT, this creates a T to C transition in comparison with the reference sequence. This technique provides two advantages over HITS-CLIP. First, the T to C transitions allow a precise identification of the Ago binding site on target RNAs. Second, the number of unspecific background sequences can be reduced by eliminating reads that lack the T to C transitions.

PAR-CLIP has been used successfully to characterize KSHV miRNA targets in two B-cell lines [23]. In total, in BC-1 cells 1741 candidate target mRNAs of the KSHV miRNAs were identified, and in BC-3 cells 1409 candidates. Well-established miRNA targets such as *CDKN1A* and *BACH1* were recovered in the analysis, in addition to several uncharacterized targets, including *CSNK1A1*, *ZFYVE9*, and *SOS1*. These targets and others were validated by luciferase reporter assays. Gene ontology analysis was performed on the KSHV miRNA targets to identify cellular pathways showing significant enrichment. BC-1 and BC-3 cells had similar results, with the top three pathways being regulation of transcription, intracellular signaling cascade, and protein localization in each case.

PAR-CLIP has the advantage that valuable information is gained on the binding sites for Ago. However, PAR-CLIP suffers from some of the same limitations as Ago-HITS-CLIP, in that the miRNA and mRNA datasets have to be bioinformatically assembled to identify miRNA–mRNA pairs. As stated above, besides assumptions about the importance of the seed sequence, the method has focused on binding sites located only within the 3′UTR of the mRNA target. There are currently indications that other types of interactions can have biological significance [12,24,25,26,27]. Finally, while both HITS-CLIP and PAR-CLIP have produced rich datasets that have led to numerous follow-up studies on specific targets, there are some additional limitations. It is important to note that both the overlap between biological replicates, as well as between HITS-CLIP and PAR-CLIP datasets, is relatively modest, suggesting that under the current conditions these assays are not comprehensive but rather sample miRNA targetomes [12,21]. The latter may reflect the assay limitations, as well as differences in individual protocols and bioinformatics data analysis parameters.

## 5. CLASH

Recently, Helwak et al. [28] developed cross-linking and sequencing of hybrids (CLASH), a method for the identification of miRNA targets which builds on the HITS-CLIP approach (Figure 1). CLASH adds an RNA ligation step prior to immunoprecipitation of the Ago protein. After RNase trimming, the ends of miRNAs and mRNA fragments that are bound to the same Ago molecule within RISC are joined to become a single “chimeric” or “hybrid” RNA molecule. This ligation step removes the ambiguity of bioinformatically annotating miRNA binding sites on mRNAs as performed in HITS-CLIP and PAR-CLIP, and generates fewer nonspecific targets and more high confidence targets [26]. Sequencing hybrids directly shows which miRNAs are bound to a specific mRNA fragment, obviating the need to connect the two by bioinformatic means. As a result, CLASH dispenses with predictions based on seed sequence that were part of the limitations of the previous methods, and generates miRNA targetomes in an unbiased manner with respect to both target interaction assumption and target RNA species.

Helwak et al. made several important findings by applying CLASH to HEK293 cells. In the first report of CLASH, rather than immunoprecipitating native Ago, as in Ago-HITS-CLIP, a tagged version of Ago was ectopically expressed. The total number of miRNA-mRNA interactions found was 18,000. The seed sequence of a miRNA consists of nucleotides 2–8 from the 5′ end, and this has been considered the primary determinant of specificity. However, a full 60% of the miRNA-mRNA interactions were found to be non-canonical, i.e., did not have a perfect seed sequence match but rather contained mismatched or bulged nucleotides. 18% of interactions did not demonstrate binding at the 5′ end, but did show base pairing at the 3′ end. 

The validity of CLASH was shown in several ways. Previously identified targets were recovered in the new dataset. Additionally, when targets found in hybrids were compared to single reads from the same dataset, 94% of the former overlapped with the latter. Published data based on the depletion of 25 different miRNAs were analyzed, and in many cases the abundance of putative target RNAs increased, consistent with the miRNA–mRNA pairs identified from CLASH. Luciferase assays were performed with sequences from hybrids containing miR-92a target sites that were both canonical and non-canonical. They found that luciferase reporter expression was upregulated in both cases when miR-92a was depleted, indicating that binding via non-canonical interactions was functional. When binding across specific miRNAs was investigated, individual miRNAs had distinct patterns of base pairing with target mRNAs.

A CLASH-like approach including RNA–RNA ligation, iPAR-CLIP, was used for analysis of interactions between miRNAs and their targets in *C. elegans* [26]. Like PAR-CLIP, 4-thiouridine was introduced prior to crosslinking, and Ago antibody was used to IP Ago-RNA complexes. The resulting sequence data had the T to C transitions, which indicate the location on the target RNA of Ago binding. They were able to identify approximately 3600 hybrids that included *C. elegans* miRNAs and mRNAs. The characteristics of the interactions were investigated. 89% of the chimeras matched Ago-binding sites as identified by the T to C transitions. The target site was located within the 3′UTR in the majority of cases. When the seed sequence matches were examined and categorized, 80% fell into the following three categories: nt 2–7 with no mismatches, nt 2–7 with one mismatch, and nt 2–8 with 2 mismatches. The remainder did not fall into any of these categories, and included non-canonical binding.

Surprisingly, Grosswendt et al. [26] found when analyzing control samples lacking the ligation step that chimeric sequences were present, although at a very low frequency. Presumably these arose from the activity of endogenous RNA ligases within cells. Based on this observation, they reanalyzed seven published HITS-CLIP and PAR-CLIP datasets, and uncovered several miRNA–mRNA interactions. Two of the datasets involved cells infected with either EBV [29] or with KSHV or KSHV plus EBV [23]. The combined total of viral miRNA-containing hybrids identified was 734. Further analysis of these hybrids suggested that many viral miRNAs do not bind to 3′UTRs and do not always interact with RNAs via seed sequence matches. In summary, the development of CLASH and the realization that hybrids can be detected in HITS-CLIP and PAR-CLIP data greatly aided the discovery of novel putative γ-herpesvirus miRNA targets.

A variant of CLASH termed CLEAR-CLIP (covalent ligation of endogenous Argonaute-bound RNAs-CLIP) has recently been described [24]. This work, with mouse brain and human hepatoma cells, also found many target sites lacking canonical binding, and underscored the importance of 3′-end pairing in miRNA/mRNA interactions.

CLASH is an extremely valuable method, but it is not without its limitations. In the original experiment, double-tagged Ago was pulled down in two separate purification steps. The more stringent purification of Ago complexes was shown to decrease the background of the experiment. When crosslinked human cell lysate was mixed with an equal amount of yeast lysate, yeast sequences were found in less than two percent of single and chimeric reads using the standard protocol. In contrast, when the second pulldown step was removed, yeast reads jumped to ten percent [25]. While it is certainly preferable to avoid background post-lysis associations [30], one must also consider that Ago overexpression may create a RISC landscape different from that seen with endogenous cellular interactions.

Another potential problem with CLASH is common to all CLIP-based protocols. To separate the Ago complexes from nonspecific proteins and free RNAs, samples are subjected to sodium dodecyl sulfate-polyacrylamide gel electrophoresis (SDS-PAGE) followed by transfer to a nitrocellulose membrane [15]. An unfortunate consequence is that many of the target Ago-RNA complexes are lost during the process. This inefficient recovery step, together with the inherently inefficient UV crosslinking and the fact that only a very small proportion of RISC-associated RNAs undergo intermolecular ligation, requires a large number of cells (from approximately 100 million [25] up to 400 million [31]) in order to obtain sufficient amounts of RNA for library construction. Hence, when fewer cells are available, or cell lines and biological samples cannot be modified by Ago-tagging, CLASH becomes quite impractical.

## 6. qCLASH

While CLASH is an improvement upon HITS-CLIP, both methods contain several post-immunoprecipitation cleanup steps resulting in large losses of RNA and as a result require large numbers of cells as input. Our goal was to modify the CLASH protocol in a way that is more amenable to small sample size and to allow recovery of hybrids from endogenous Ago.

**Figure 2:**
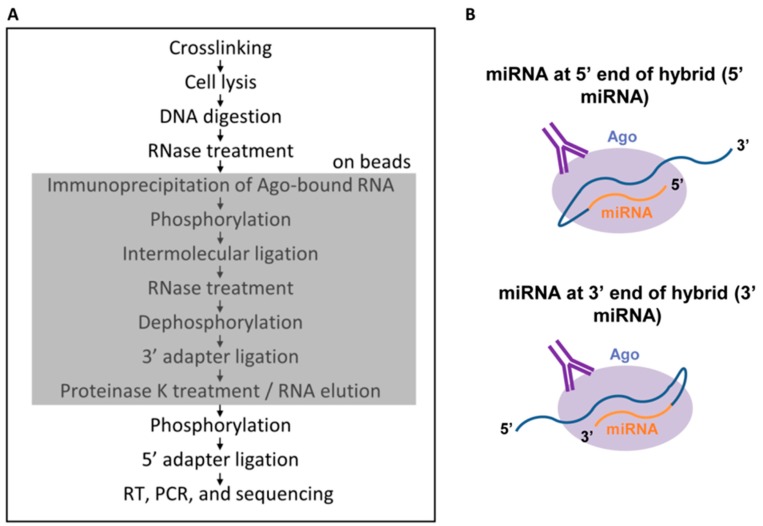
(**A**) The quick CLASH (qCLASH) method. The shaded box indicates the portion of the method that takes place on beads; (**B**) The two possible ways in which microRNAs (miRNAs) and mRNAs can join during intermolecular ligation. From [27], Copyright © 2018 American Society for Microbiology, *Journal of Virology*, 92, e02138-17, 2018.

In the original CLASH protocol [25], the ligation step which gives rise to hybrids is performed prior to the cleanup steps. We reasoned that, given current increased sequencing capabilities, in combination with the relatively straightforward bioinformatic steps to identify hybrids, it was not necessary to perform clean-up steps. Extraneous RNAs and hybrids could all be sequenced and the non-hybrids could simply be ignored. In keeping with this line of thinking, we have developed a shortened CLASH protocol [27], which omits the steps resulting in the largest losses of RNA, the gel purification and transfer and elution from a membrane. We also perform all steps on beads to prevent any precipitations, which introduce salt that negatively impacts linker ligation efficiency (Figure 2A). The miRNA and mRNA can be ligated in either of two possible orientations, with the miRNA at the 5′ end of the hybrid RNA or at the 3′ end (Figure 2B). We have named the new protocol quick CLASH, or qCLASH, as it takes significantly less time than the original method. It can also be completed using fewer cells as input, making it preferable for cell types which are difficult to grow in large quantities and for clinical samples. The lowest number of cells we have successfully performed qCLASH with is 10 million (in preparation).

We performed the first qCLASH study to identify the KSHV miRNA targetome in endothelial cells [27]. While the most important KSHV-associated cancer is Kaposi’s sarcoma, thus far, all ribonomics studies to identify miRNA targets have utilized PEL cells as described above. The cells used were TIVE-EX-LTC, a fast-growing derivative of TIVE-LTC (telomerase-immortalized vein endothelial long-term culture cells) that had lost the KSHV genome and can be reinfected with KSHV [27]. TIVE-EX-LTC cells were either uninfected or latently infected with either wild-type KSHV or with a recombinant BAC16-derived virus lacking miR-K12-11/11* [32] as a specificity control, with three bioreplicates of each. Following the qCLASH procedure and the identification of hybrid RNAs by the Hyb program [33], the proportion of sequencing reads that were hybrids was between 0.3% and 1%. Of these, a subset were miRNA–mRNA hybrids, and a further subset were KSHV miRNA-mRNA hybrids. Despite the relatively low proportion of reads that fulfilled these criteria, the numbers of sequencing reads were high enough to enable recovery of approximately 6000 to 12,000 hybrids between KSHV miRNAs and cellular mRNAs per wild type biological replicate. The initial analysis focused only on hybrids between miRNAs and cellular mRNAs [27]. 95% of the miRNA-mRNA hybrids had the miRNA located at the 5′ end of the hybrid (Figure 2B), with the remaining 5% having the miRNA at the 3′ end. 

The data allowed a comparison of the properties of both cellular miRNAs and KSHV miRNAs in targeting mRNAs. Surprisingly, miRNAs bound to mRNAs within the CDS in >50% of observed hybrids. For cellular miRNAs, the proportion of miRNA target sites that were located within the CDS was 52%, and for viral miRNAs 65%. As with earlier CLASH studies, a high frequency of base pairing toward the 3′ end of the miRNA, outside of the seed sequence, was observed for both viral and cellular miRNAs. A recent study [34] showed that miRNA target sites within the CDS repress translation but do not affect mRNA stability. These miRNA-recognition elements do require extensive miRNA 3′ base pairing; however, only minimal seed sequence pairing is needed. miRNA target sites in the CDS require Ago to reduce protein levels but not GW182, a RISC partner that leads to mRNA decay. 

**Figure 3:**
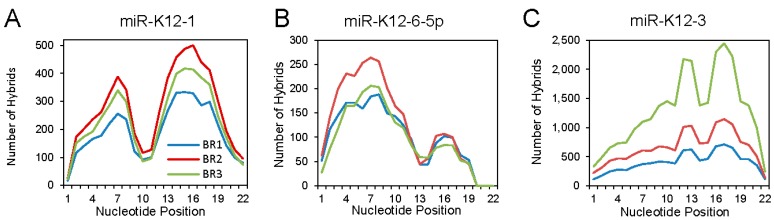
Patterns of binding for selected individual Kaposi’s sarcoma-associated herpesvirus (KSHV) miRNAs. The status of each nucleotide (bound or unbound) along the length of the miRNA portion of each hybrid was determined, based on the Vienna diagrams generated through Hyb. BR1, BR2 and BR3 indicates biological replicates 1, 2 and 3. (**A**) KSHV miR-K12-1; (**B**) KSHV miR-K12-6-5p; (**C**) KSHV miR-K12-3. Adapted from [27], Copyright © 2018 American Society for Microbiology, *Journal of Virology*, 92, e02138-17, 2018.

Analyzing the binding interactions of individual KSHV miRNAs revealed different binding patterns that seemed to be miRNA specific. Three examples are shown in Figure 3. For miR-K12-1, an M-shaped plot was found, with aggregate binding extending over the seed sequence and the 3′ region. For miR-K12-6-5p, mainly canonical binding was seen. At the other extreme, miR-K12-3 showed mostly 3′ interaction: this unusual pattern was noted previously [26].

We next analyzed cellular and viral miRNA–mRNA interactions that lacked a strong canonical seed sequence. For cellular miRNAs, 46% of the interactions fell into the non-canonical category, and for viral miRNAs, 56%. These data are in agreement with previous CLASH studies and also show that there is not a large difference between viral and cellular host miRNA targeting. Importantly, miRNA targeting within the CDS, as well as an expanded pool of 3′ interactions, could not be discovered by previous techniques. qCLASH has significantly expanded the putative KSHV miRNA targetome.

Analyzing the hybrids in WT infected cells identified 54 putative high-confidence miR-K12-11 targets, none of which were recovered from cells infected with the mutant lacking miR-K12-11. This suggested that qCLASH had no non-specific background, at least for this miRNA. We validated some of these targets by transfecting a miR-K12-11 mimic into TIVE-EX-LTC cells infected with KSHV lacking miR-K12-11/11*, and comparing transcript levels with cells transfected with a non-targeting control mimic. 26 of 40 tested genes showed a significant reduction of mRNA level with the miR-K12-11 mimic, and this set included genes where the miRNA binding site resides within the CDS.

Pathway analysis with Ingenuity of the 1433 putative miRNA targets that appeared in 3/3 biological replicates from cells infected with WT KSHV yielded several pathways that were enriched for multiple targets. Some of the major pathways found were apoptosis, cell cycle control, and glycolysis, which are relevant to the properties of a tumorigenic γ-herpesvirus. The Warburg effect changing glucose metabolism was previously shown to be strongly induced in KSHV-infected cells [35].

## 7. Interactions of microRNAs with lncRNA Targets

The original published studies of miRNA targeting using HITS-CLIP and PAR-CLIP did not take into account the possibility of miRNAs targeting other RNAs including lncRNAs. Since that time, de-regulation of lncRNAs in cancer has become a new hallmark of many human malignancies [36,37,38]. Although several lncRNAs have been identified whose expression is perturbed in multiple malignancies, only a small proportion of these are understood at a mechanistic level. Both computational [39] and experimental [40,41] studies have provided evidence that miRNAs can target lncRNAs, with biological consequences. A recent study of KSHV-infected TIVE cells [13] used wild-type and miRNA-deleted KSHV in conjunction with microarray technology to profile lncRNA expression. 126 host lncRNAs were identified as putative targets of KSHV miRNAs. KSHV was found to deregulate hundreds of host lncRNAs, including the cancer-associated lncRNAs UCA1, ANRIL and MEG3. KSHV miRNAs were shown by biochemical means to bind directly to host lncRNAs, and transfection with microRNA mimics reduced the level of specific lncRNAs, including ANRIL and MEG3. These data established that KSHV de-regulates lncRNA in a miRNA-dependent fashion.

A systematic re-analysis of published HITS-CLIP, PAR-CLIP and qCLASH data was undertaken to identify computationally lncRNA targets of KSHV and EBV miRNAs [12]. NCBI-deposited raw data from HITS-CLIP and PAR-CLIP assays were obtained from PEL cells infected with KSHV or from B cells transformed with EBV [21,23,29,42,43]. Sequence reads were aligned to the human genome (hg19) and fed into the PIPE-CLIP pipeline. When all the potential targets for viral miRNAs were pooled across all CLIP datasets, the proportion of targets falling into the mRNA category was 61%, while lncRNAs comprised 35% of total targets. lncRNAs therefore represent a substantial portion of EBV and KSHV miRNA targets. In terms of numbers, viral miRNAs targeted hundreds of cellular lncRNAs. Importantly, this analysis also identified cellular miRNAs targeting thousands of lncRNAs, indicating that lncRNA targeting by miRNAs is not a peculiarity of viral miRNAs. Some (99) of the lncRNA targets of KSHV and EBV were reported to be aberrantly expressed in cancer. However, given the relative infancy of the lncRNA field, this number is likely to be an underestimate. 

To directly analyze viral and cellular miRNA–lncRNA interactions, we also analyzed the above described qCLASH data set from endothelial cells infected with either wild-type KSHV or with the miR-K12-11 mutant. However, in this case seed sequence prediction was not required since only hybrid molecules were analyzed. The numbers of lncRNA hybrids with KSHV miRNAs found varied between biological replicates from 691 to 4223. For lncRNA hybrids with cellular miRNAs, the numbers ranged from to 14,269 to 44,144. As before, some of the cellular targets of KSHV miR-K12-11 were validated by transfecting TIVE-EX-LTC cells with a miR-K12-11 mimic or with a control mimic and then measuring the amount of lncRNA by RT-qPCR. Of 13 potential targets with measurable transcript levels, nine gave decreased expression with the miR-K12-11 mimic. These data showed that miRNA targeting of lncRNA, like mRNA, can induce RNA turnover. An additional hypothesis might be that some lncRNA–miRNA interactions reflect the opposite, where lncRNAs act as competing endogenous RNAs for miRNAs (also called sponging) [44]. 

The properties of miRNA–lncRNA interactions were compared with those of miRNA–mRNA interactions. For lncRNA targets, defining the miRNA target location as being within the CDS or 3′UTR is obviously not possible. Instead the lncRNA targets were divided into 5 segments, and the proportion of binding sites falling within regions 0–20%, 20–40%, 40–60% etc. of the lncRNA was plotted [12]. A similar analysis was performed for mRNA hybrids. For hybrids with the miRNA at the 5′ end of the chimeric RNA (Figure 2B), a uniform distribution of binding sites along the length of the lncRNA was observed, and this was similar for mRNAs. However, for hybrids with the miRNA at the 3′ end, for lncRNA targets, there was a significant bias toward binding at the 3′ end of the target (regions 60–80% and 80–100%). A similar bias was not seen for 3′ hybrids involving mRNAs. The structures of RNAs, their association with proteins, and their flexibility presumably all play a role in determining if a free end is available for ligation to a miRNA within RISC.

The dependence of miRNA–lncRNA interactions on seed sequence pairing was investigated by categorizing the events into binding over nucleotides 2–8 (0, 1 and 2 mismatches); binding for nt. 2–7 (no mismatches); and “other”. The extent of non-canonical binding with lncRNA targets was >50% for both cellular and viral miRNAs, and was slightly higher than the extent seen with mRNA targets. When the binding profiles of individual KSHV miRNAs were plotted and compared for lncRNA targets vs. mRNA targets, the profiles were strikingly similar, for example for the strong seed sequence dependent miR-K12-6-5p, and for the strong non-canonical binding of mIR-K12-3. The nature of the target (lncRNA or mRNA) did not influence the binding patterns of the individual miRNAs that were studied but instead the miRNA itself dictated seed-sequence dependence.

The significance of the many miRNA–lncRNA interactions is not clear at this point. Although some miRNA–lncRNA interactions led to RNA turnover, others did not. The consequences of these interactions may involve perturbing lncRNA–protein binding that is required for lncRNA functioning. However, until the functions of more lncRNAs have been mechanistically determined, this remains a difficult question to answer. Another possibility is that lncRNAs could function as miRNA sponges (or competing endogenous RNAs) [44], thus derepressing mRNAs that would otherwise be inhibited. Systems biology approaches will ultimately be needed to provide a better assessment of this scenario.

## 8. Conclusions

**Ribonomics data accelerates our understanding of miRNAs in viral cancer**: The rapid development and evolution of innovative ribonomics approaches to determine global miRNA targetomes has enabled study of how miRNAs, and here specifically miRNAs expressed by the tumorigenic γ-herpesviruses EBV and KSHV, shape host cell transcriptomes. Several detailed molecular studies have already arisen from target catalogs created by HITS-CLIP and PAR-CLIP datasets and new ones will follow utilizing qCLASH datasets that have been generated for KSHV and are in progress for additional herpesviruses. These studies have also been aided by a large number of recombinant viruses lacking specific miRNAs.

**Unbiased CLASH approaches expand miRNA targetomes**: Both CLASH and qCLASH studies showed more CDS binding by miRNAs and more non-canonical interactions. As a result, thousands of additional putative miRNA targets have been identified that were missed using the initial HITS-CLIP and PAR-CLIP approaches that were driven by bioinformatic assumptions. Recently, an alternative mechanism has been proposed by which Ago complexes interact with miRNA target sites within the CDS versus 3′UTR, and it will be interesting to see if similar differences exist for lncRNA targeting.

**MiRNAs target lncRNAs and can induce lncRNA turnover**: Using bioinformatics pipelines to analyze HITS-CLIP and PAR-CLIP data sets as well as qCLASH experimental data identified thousands of viral miRNA–lncRNA and cellular miRNA–lncRNA interactions. Moreover, comparing binding properties of miRNA–mRNA vs miRNA–lncRNA did not reveal any significant differences. Although the exact molecular consequences of these putative miRNA–lncRNA interactions are currently not well understood, they represent a novel paradigm of gene regulation and warrant further study. 

**Where to go from here?** As with so many contemporary genomics research, all of these findings greatly increase the overall complexity of biological systems. One may argue that there will not be simple ways to generate further insight into how miRNA–lncRNA interactions may cause biologically significant effects. On the contrary, we suggest that applying systems biology and machine learning approaches to comparatively analyze CLASH, RNAseq, proteomics and metabolomics datasets with a focus on miRNA/lncRNA interactions could provide an opportunity to functionally annotate hitherto uncharacterized lncRNAs.
